# Deficiency of ribosomal protein S26, which is mutated in a subset of patients with Diamond Blackfan anemia, impairs erythroid differentiation

**DOI:** 10.3389/fgene.2022.1045236

**Published:** 2022-12-12

**Authors:** Noemy Piantanida, Marta La Vecchia, Marika Sculco, Maria Talmon, Gioele Palattella, Ryo Kurita, Yukio Nakamura, Antonella Ellena Ronchi, Irma Dianzani, Steven R. Ellis, Luigia Grazia Fresu, Anna Aspesi

**Affiliations:** ^1^ Department of Health Sciences, Università Del Piemonte Orientale, Novara, Italy; ^2^ Department of Research and Development, Central Blood Institute, Blood Service Headquarters, Japanese Red Cross Society, Tokyo, Japan; ^3^ Cell Engineering Division, RIKEN BioResource Research Center, Tsukuba, Japan; ^4^ Department of Biotechnology and Biosciences, University of Milano-Bicocca, Milan, Italy; ^5^ Department of Biochemistry and Molecular Genetics, University of Louisville, Louisville, KY, United States

**Keywords:** ribosomal protein, Diamond Blackfan anemia, ribosomopathy, RPS26, erythroid differentiation

## Abstract

**Introduction:** Diamond Blackfan anemia (DBA) is a rare congenital disease characterized by defective maturation of the erythroid progenitors in the bone marrow, for which treatment involves steroids, chronic transfusions, or hematopoietic stem cells transplantation. Diamond Blackfan anemia is caused by defective ribosome biogenesis due to heterozygous pathogenic variants in one of 19 ribosomal protein (RP) genes. The decreased number of functional ribosomes leads to the activation of pro-apoptotic pathways and to the reduced translation of key genes for erythropoiesis.

**Results and discussion:** Here we characterized the phenotype of RPS26-deficiency in a cell line derived from human umbilical cord blood erythroid progenitors (HUDEP-1 cells). This model recapitulates cellular hallmarks of Diamond Blackfan anemia including: imbalanced production of ribosomal RNAs, upregulation of pro-apoptotic genes and reduced viability, and shows increased levels of intracellular calcium. Evaluation of the expression of erythroid markers revealed the impairment of erythroid differentiation in RPS26-silenced cells compared to control cells.

**Conclusions**: In conclusion, for the first time we assessed the effect of RPS26 deficiency in a human erythroid progenitor cell line and demonstrated that these cells can be used as a scalable model system to study aspects of DBA pathophysiology that have been refractory to detailed investigation because of the paucity of specific cell types affected in this disorder.

## 1 Introduction

Diamond Blackfan anemia (DBA) is an inherited bone marrow failure syndrome that classically presents with severe anemia at birth, when fetal hemoglobin (HbF) is predominant, or shortly afterward (Vlachos and Muir, 2010). The diagnosis is established at a median age of 2–3 months (Costa et al., 2020), and the incidence is of about seven per million live births (Vlachos et al., 2008). DBA is due to the inability of erythroid progenitors to proceed with erythroid maturation, while the other bone marrow cell lineages generally show normal counts. The clinical manifestations are heterogeneous and include red cell aplasia, congenital malformations and cancer predisposition, both to hematological malignancies and solid tumors (Vlachos et al., 2018; Lipton et al., 2022). Corticosteroids are a first-line therapy for the anemia in DBA and are effective in at least half the cases. Steroid-resistant patients are treated with chronic transfusions and iron chelation, or by hematopoietic stem cell transplantation (Vlachos and Muir, 2010).

In most cases, DBA is an autosomal dominant disease caused by loss-of-function heterozygous variants in one of 19 ribosomal protein (RP) genes, that encode for proteins of both the small (*RPS19*, *RPS26*, *RPS10*, *RPS24*, *RPS17*, *RPS7*, *RPS27*, *RPS29*, *RPS28*, *RPS15A*) and the large (*RPL5*, *RPL11*, *RPL35A*, *RPL26*, *RPL15*, *RPL31*, *RPL27*, *RPL35*, *RPL18)* ribosomal subunit (Ulirsch et al., 2018). *RPS19* was the first DBA gene identified and is by far the most frequently mutated gene in DBA patients (Boria et al., 2010). RPS19 is required for 18S ribosomal RNA (rRNA) maturation and for the formation of 40S ribosomal subunits (Flygare et al., 2007). Its haploinsufficiency results in p53 accumulation and cell cycle arrest in human hematopoietic progenitor cells (Dutt et al., 2011). In *rps19*-deficient zebrafish and mice, defective hematopoiesis can be alleviated by suppression of p53 (Danilova et al., 2008; Jaako et al., 2015).

While there is compelling evidence implicating p53 activation in DBA pathophysiology, it does not provide a ready explanation for the tissue selectivity of clinical phenotypes. Downstream effects of defects in ribosome biogenesis on the translational output have been linked to the red cell hypoplasia in DBA patients. For example, free heme resulting from the dysregulation of the balance between heme synthesis and globin expression subconsequent to a general reduction of protein synthesis, is expected to contribute to DBA pathophysiology, since free heme is highly toxic (Keel et al., 2008; Doty et al., 2015; Mercurio et al., 2016). Alternatively, it has been shown that the limited ribosome content not only affects global translation (Cmejlova et al., 2006), but also preferentially alters the translation of *GATA1* mRNA, which encodes for a key hematopoietic transcription factor, and possibly of other erythroid mRNAs (Ludwig et al., 2014).

While many of the studies have focused initially on RPS19, other ribosomal proteins, notably RPL5 and RPL11 have also been characterized providing additional support for the mechanisms outlined above (Moniz et al., 2012; Garçon et al., 2013). Here, we turn to another ribosomal protein affected in DBA patients, RPS26. The *RPS26* gene is one of the most frequently mutated gene in DBA patients and is found mutated in 5.3–-11.6% of cases (Doherty et al., 2010; Smetanina et al., 2015; van Dooijeweert et al., 2018; Quarello et al., 2020; Volejnikova et al., 2020; Lipton et al., 2022). RPS26, like other ribosomal proteins affected in DBA, is required for ribosome biogenesis, specifically for the maturation of 40S ribosomal subunits. RPS26 has some unusual properties that are not shared by other proteins affected in DBA. Studies in yeast have shown that Rps26 may be able to dissociate from mature 40S subunits and give rise to a population of Rps26-deficient subunits with distinct properties in translation (Ferretti et al., 2017; Yang and Karbstein, 2022). These distinctive properties of Rps26 may give rise to some of the phenotypic characteristics observed in DBA patients with RPS26 mutations, specifically, the fact that no RPS26-mutated patient has developed myelodysplastic syndrome or cancer, so far (Lipton et al., 2022). For these reasons we sought to establish a scalable model system for studying the effects of RPS26 deficiency in a setting that recapitulates salient features of DBA.

## 2 Materials and methods

### 2.1 Cell cultures and *RPS26* silencing

HUDEP-1 cells are an immortalized cell line able to produce enucleated erythroid cells, which was derived from CD34-positive cells isolated from umbilical cord blood. These cells were obtained by transduction with a HPV16-E6/E7 expression system that is induced by doxycycline (Kurita et al., 2013). HUDEP-1 cells express mainly HbF.

Cells were cultured in Expansion Medium (EM) in the presence of 3 µM doxycycline (DOX). EM was composed of StemSpan^®^ SFEM (Stemcell Technologies), 75 ng/ml stem cell factor (SCF), 2U/mL erythropoietin (EPO) and 1 µM dexamethasone. For induction of erythroid differentiation, cells were cultured for 2 days in Differentiation Medium (DM) i.e. Iscove’s Modified Dulbecco’s Medium (IMDM) supplemented with 2% fetal bovine serum, 3% human serum, 3 U/mL heparin, 10 μg/ml insulin, 330 μg/ml human holo-transferrin and 3 U/mL EPO. Before and after each experiment, cell count and viability were determined using the trypan blue dye exclusion test.

For *RPS26* silencing, 10^6^ HUDEP-1 cells were nucleofected with 50 pmol of either RPS26-specific or negative control siRNA using the P3 Primary Cell 4D-Nucleofector™ X Kit and the 4D-Nucleofector^®^ system (Lonza) (program EE-100). Three Silencer^®^ Select siRNAs (Ambion) were used to downregulate *RPS26* expression (IDs: s199002 siRNA-A, s229469 siRNA-B, s197282 siRNA-C), whereas a Silencer^®^ Negative Control siRNA (Ambion), designed to avoid interactions with human transcripts, was used as negative control. Nucleofected cells were cultured in EM for 4 days and *RPS26* knockdown was evaluated by real-time PCR and western blot. For experiments where erythroid differentiation was induced, nucleofected cells were cultured first in EM for 2 days, and then in DM for two more days.

### 2.2 Quantitative RT-PCR (qRT-PCR)

Total RNA for qRT-PCR analysis was isolated using TRIzol™ reagent (Invitrogen) followed by DNase treatment and purification with miRNeasy Mini Kit (Qiagen). cDNA was synthesized using the High Capacity cDNA Reverse Transcription Kit (Applied Biosystems). Quantitative PCR was performed on a CFX96 Real-Time PCR Detection System (Biorad) using either Taqman^®^ Gene Expression Assays (Applied Biosystems) or Power SYBR Green PCR master mix (Applied Biosystems). Primer sequences used for qRT-PCR are available upon request. PCR reactions were run in triplicate. Threshold cycle (Ct) values were normalized to *GAPDH*, used as endogenous control, and expression levels were calculated using formula 2^-ΔCt, where ΔCt = Ct_target gene_—Ct_reference gene_.

### 2.3 Western blot

Cells were washed with Phosphate Buffered Saline (PBS) and lysed as previously described (Aspesi et al., 2014). Proteins were separated by electrophoresis on a 12% SDS-PAGE gel, transferred to nitrocellulose membranes and incubated with antibodies specific for RPS26 (#SAB1302676, Sigma-Aldrich), alpha globin (#sc-514378, Santa Cruz Biotechnology), gamma globin (#sc-21756, Santa Cruz Biotechnology) and GAPDH (#2118, Cell Signaling). Band intensities were quantified by densitometry in ImageJ.

### 2.4 rRNA analysis

To study mature rRNAs, total RNA was analyzed using the RNA 6000 Nano Kit (Agilent Technologies) on the Agilent 2100 Bioanalyzer system according to the manufacturer’s protocol. RNA integrity number (RIN) and 28S/18S ratio were acquired for each RNA sample.

### 2.5 Flow cytometry

To detect apoptotic cells with exposed phosphatidylserine, cells were incubated for 30 min with FITC-conjugated Annexin-V (#31490013, Immunotools) and analyzed using FACSCalibur flow cytometer (BD Biosciences). For cell cycle analysis, cells were fixed in 70% cold ethanol, washed with PBS, treated with RNase A, and stained for 30 min with 40 μg/ml propidium iodide in the dark, at room temperature. DNA content was analyzed by measuring fluorescence intensity through flow cytometry. For analysis of membrane erythroid markers, cells were stained for 30 min with FITC conjugated anti-CD235a (Glycophorin A) antibody (#559943 BD Biosciences) and APC conjugated anti-CD71 (#551374 BD Biosciences). Fluorescent signals were measured using FACSCalibur.

### 2.6 Calcium level measurement

To evaluate Ca^2+^ concentrations in the cytosol we used a custom built aequorinometer (CAIRN Research). 50,000 cells were transduced by a third-generation lentiviral vector (LV) carrying the native aequorin (cytAEQ-LV). Plasmids and lentiviral particles were generated as already reported (Talmon et al., 2019). After 72 h, cells were washed with Krebs-Ringer buffer (KRB; 135 mM NaCl, 5 mM KCl, 0.4 mM KH_2_PO_4_, 1 mM MgSO_4_, 5.5 mM glucose, 20 mM HEPES, pH 7.2) and reconstituted with native coelenterazine (#C2230, Sigma-Aldrich) at 37°C, in the dark for 30 min. The cells were transferred into perfusion chamber of the aequorinometer in KRB-Ca^2+^ and the baseline recorded for 100 s. Then, cells were stimulated with ATP 100 µM. For quantification of Ca^2+^ concentration, at the end of each experiment cells were perfused with distilled water containing 0.1% Triton and 50 mM Ca^2+^ to discharge the remaining aequorin pool. Emitted light was converted in Ca^2+^ concentrations offline using a previously described algorithm (Brini et al., 1995). All measurements were carried out at 37°C.

### 2.7 Statistical analysis

Data are presented as mean ± standard error of the mean. The assumption of Gaussian distribution was not possible due to the small sample size of biological replicates (N = 3–6), therefore a non-parametric test, i.e. the Mann-Whitney test, was used to determine differences between datasets (Nahm, 2016). Comparisons were performed using GraphPad Prism five software. A *p*-value ≤0.05 was considered statistically significant.

## 3 Results

### 3.1 *RPS26* silencing alters 28S/18S rRNA ratio in HUDEP-1 cells

**FIGURE 1:**
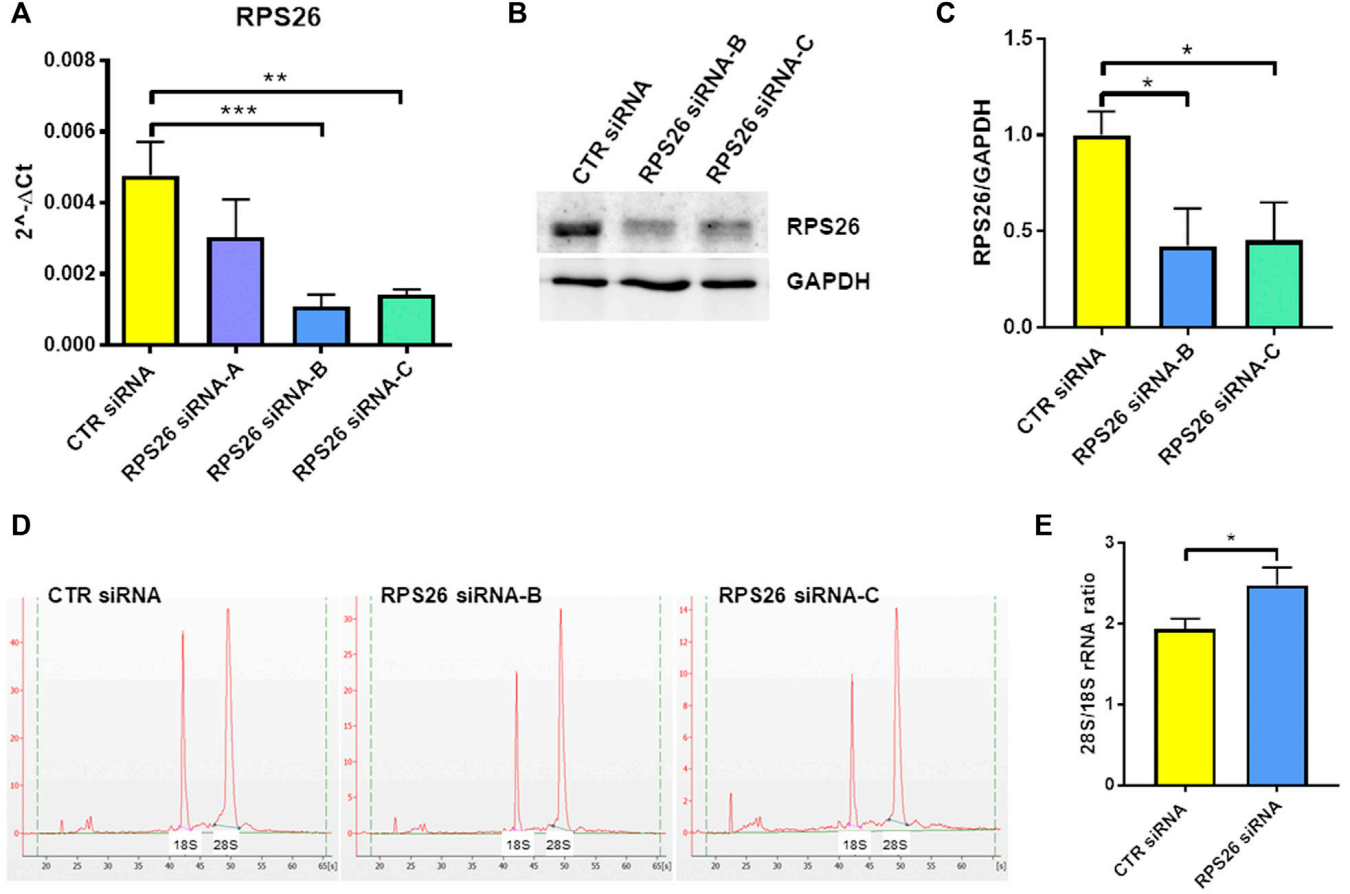
RPS26 silencing in HUDEP-1 cells. **(A)** Quantitative RT-PCR to measure *RPS26* transcript was performed on RNA isolated from cells 4 days after nucleofection with a control (CTR) or three RPS26-specific siRNAs **(A,B and C)**. *RPS26* levels were normalized to *GAPDH*; error bars represent standard error of the mean (SEM), **p-value ≤ 0.01, ***p ≤ 0.001. **(B)** Representative immunoblot performed on nucleofected cells. **(C)** Densitometric analysis of three immunoblots performed on nucleofected cells; the expression of RPS26 was normalized to the housekeeping gene, *GAPDH*. Bars represent the mean ± standard deviation (SD), *p ≤ 0.05. **(D)** Representative images of total RNAs isolated from control and RPS26-deficient cells and analyzed by the Agilent 2100 Bioanalyzer. **(E)** Bioanalyzer results expressed as the ratio of 28S/18S mature rRNAs: graph bars represent the mean ± SD of two experiments performed with RPS26 siRNA-B and two experiments performed with RPS26 siRNA-C, *p ≤ 0.05.

To specifically downregulate RPS26 in HUDEP-1 cells, we performed nucleofection using a 4D-nucleofector system (Lonza) and three different small interfering RNA (siRNA) targeting *RPS26* transcript (siRNA S26-A, B and C). Cells were harvested 4 days after transfection to extract total RNA and perform qRT-PCR for *RPS26*. Based on our previous experience on RPS19 downregulation (Aspesi et al., 2014) (and unpublished data), we knew that a strong silencing of the RP transcript is required in cell models to mimic the RP haploinsufficiency observed in DBA patients. Therefore, for the subsequent experiments we only used siRNA S26-B and C, which were the most effective (Figure 1A) and reduced RPS26 protein levels by about 50%, as shown by western blot analysis (Figures 1B,C).

It is known that the deficiency of a RP located in the small or large ribosomal subunit disrupts the biogenesis or maturation of that specific subunit and leads to decreased amount of the respective mature rRNA components (Robledo et al., 2008). For example, haploinsufficiency of either *RPS19* or *RPS26* causes reduced 18S rRNA amount and therefore increased 28S/18S rRNA ratio (Flygare et al., 2007; Doherty et al., 2010). The alteration of the 28S/18S rRNA ratio can be detected by capillary electrophoresis instruments, such as the Bioanalyzer system, as demonstrated in studies performed on both mononuclear blood cells and lymphoblastic cell lines derived from DBA patients (Farrar et al., 2014; Quarello et al., 2016; Aspesi et al., 2017). Accordingly, the analysis of the total RNA isolated from RPS26-silenced HUDEP-1 cells (Figure 1D) showed a statistically significant increase (*p* < 0.05) of the 28S/18S rRNA ratio, when compared to control (Figure 1E).

### 3.2 RPS26-downregulated HUDEP-1 cells are prone to apoptosis

**FIGURE 2:**
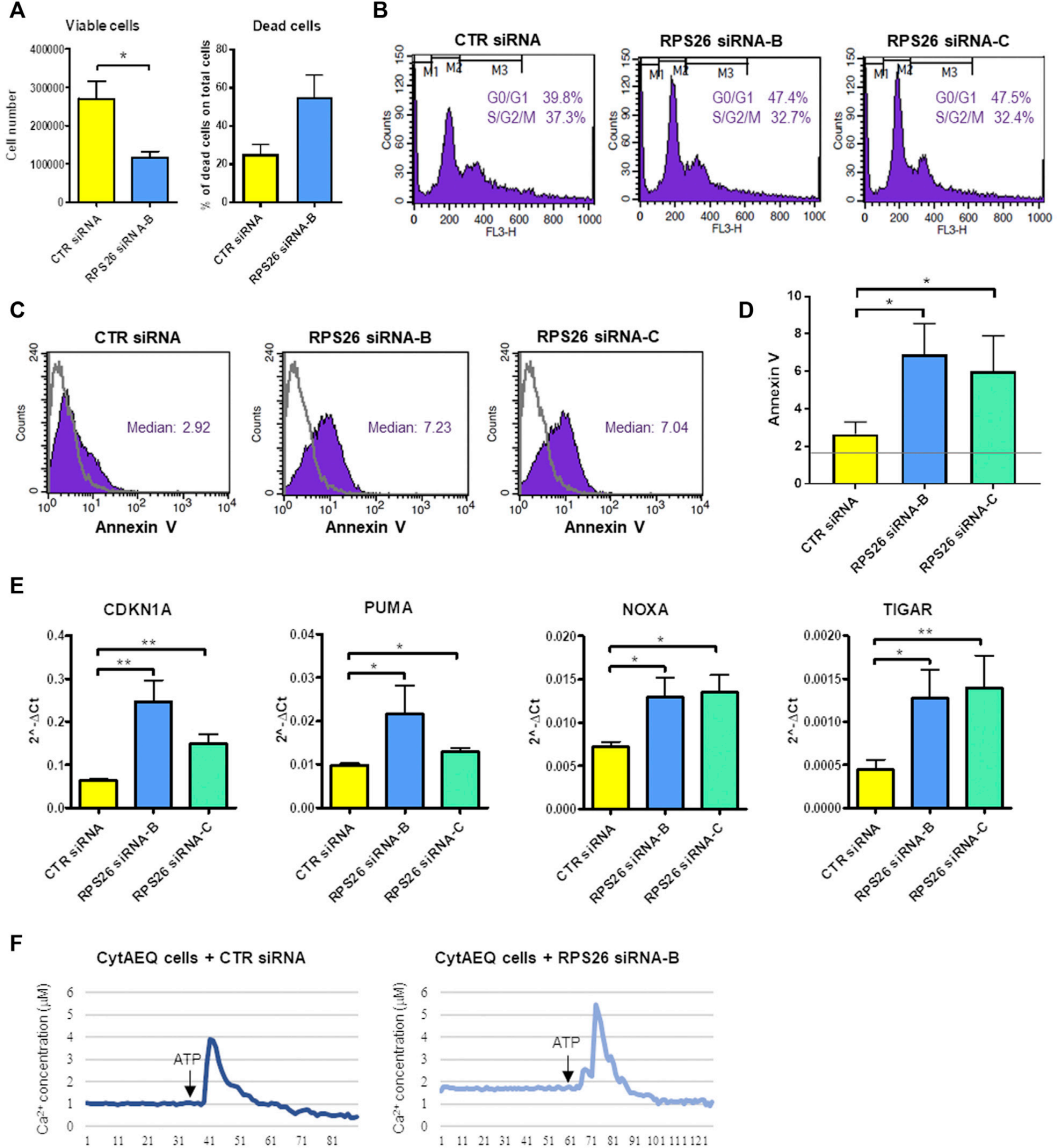
Phenotype of RPS26-downregulated HUDEP-1 cells. **(A)** Viable and dead cells were counted with trypan blue staining 4 days after nucleofection with negative control (CTR) or RPS26 specific siRNAs. Bars represent mean ± SEM calculated on three experiments, *p ≤ 0.05. **(B)** Representative cell cycle analysis by flow cytometry of control and RPS26-silenced cells stained with propidium iodide. **(C)** Representative Annexin V assay for apoptosis detection performed on control and RPS26-silenced cells. The grey line represents the unstained negative control. **(D)** Bar graph showing the mean ± SD of the median value of fluorescence of cells stained with Annexin V-FITC; results of three experiments, *p ≤ 0.05. The horizontal grey line represents the median value of fluorescence of unstained control. **(E)** Expression levels of pro-apoptotic gene targets in control and RPS26-deficient cells measured by qRT-PCR 4 days after nucleofection. Data were obtained from at least three experiments and normalized on *GAPDH* expression. Error bars represent SEM, *p ≤ 0.05, **p ≤ 0.01. **(F)** Cytosolic calcium concentration in control cells and in cells treated with RPS26 siRNA-B. Cells were transduced with a lentiviral vector to induce the expression of aequorin in the cytoplasm and calcium levels was quantified by using an aequorinometer. The horizontal axis represents time expressed in seconds.

*RPS26*-downregulated cells showed decreased proliferation and increased cell death compared to cells transfected with a negative control siRNA (Figure 2A). We analyzed the distribution of cells in different phases of cell cycle and we found that when the expression of RPS26 was reduced, HUDEP-1 cells were arrested in G0/G1 phase of the cell cycle (Figure 2B). Cells with RPS26 knockdown also showed significantly increased apoptosis compared to control cells, as measured by flow cytometric annexin V staining (Figures 2C,D). Downregulation of RPS26 in HUDEP-1 cells did not modify the expression of p53 (Supplementary Figure S1), and treatment with 25 μM pifithrin-α, a p53 inhibitor (Komarov et al., 1999), did not significantly decrease annexin V positivity of RPS26-silenced cells (data not shown), suggesting that p53 is not a major player in the induction of apoptosis in this cell model. We then checked the expression of four genes that are involved in apoptotic signalling pathways, i.e. *CDKN1A*, *PUMA*, *NOXA*, *TIGAR*, and we observed that all of them were upregulated in RPS26-deficient cells (Figure 2E). Overall, these data suggest that RPS26-deficient cells are prone to apoptosis.

Intracellular Ca^2+^ is one of the most important intracellular messengers and plays functional roles in different cell processes, such as cell cycle progression, cell differentiation and cell death (Zhang et al., 2022). We thus decided to measure the levels of calcium in the cytoplasm of RPS26-deficient and control HUDEP-1 cells. To this end, the gene coding for the calcium-binding photoprotein aequorin was transferred to HUDEP-1 cells by lentiviral transduction. The binding of aequorin to calcium is accompanied by the emission of blue light, that can be quantified by using an aequorinometer (Lim et al., 2016). We observed that the basal level of calcium was increased in cells silenced with RPS26 siRNA-B when compared to control cells (Figure 2F). The treatment for 24 h with increasing doses of the Ca^2+^ chelator EGTA (0.5–2 mM) did not alter annexin V positivity of RPS26-silenced cells (data not shown), suggesting that cell death was not due to calcium accumulation.

### 3.3 Erythroid differentiation in HUDEP-1 cells

**FIGURE 3:**
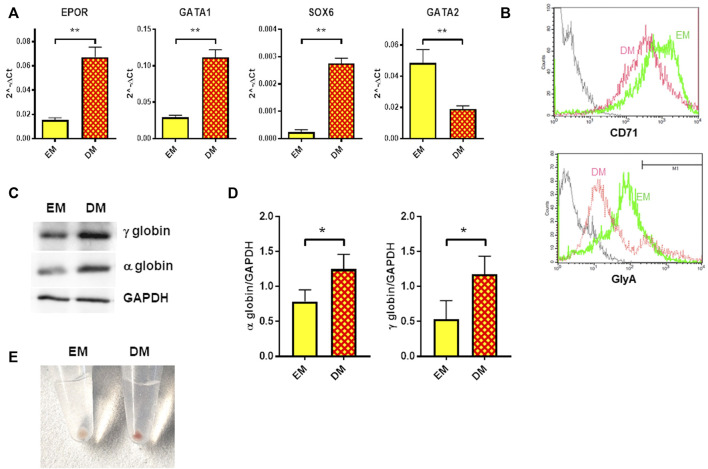
Erythroid differentiation of HUDEP-1 cells. **(A)** Expression of erythroid transcripts assessed by qRT-PCR, normalized on *GAPDH* expression; results are presented as mean ± SEM calculated on at least three experiments; **p ≤ 0.01. **(B)** Representative flow cytometry analysis of the expression of the erythroid surface markers CD71 and GlyA in cells cultured in EM (green) or DM (magenta). The grey line represents the unstained control. GlyA-positive cells, as defined by the marker M1, were 15.3% for EM-cultured cells and 17.6% for DM-cultured cells. **(C)** Representative immunoblot performed on EM- and DM-cultured cells, showing the expression levels of α- and γ-globins; GAPDH was used as loading control. **(D)** Densitometric analysis of three immunoblots performed as in Figure 3C; the expression of the globins was normalized to GAPDH. Bars represent the mean ± SD, *p ≤ 0.05. **(E)** Cells cultured for 48 h in DM produced a red pellet, upon centrifugation.

Erythroid differentiation was induced in HUDEP-1 cells by culturing them in Differentiation Medium (DM) for 48 h. In agreement with the report of Kurita and coll. (Kurita et al., 2013), we observed that HUDEP-1 cells could differentiate into more mature erythroid stages. The gene expression profile of erythroid markers evaluated by quantitative RT-PCR showed, as expected (Kurita et al., 2013), that cells cultured in DM had increased levels of *EPOR*, *GATA1* and *SOX6* transcripts and decreased *GATA2* expression, when compared to cells cultured in Expansion Medium (EM) (Figure 3A). Flow cytometry analysis after staining for the erythroid markers CD71 (transferrin receptor) and CD235a (glycophorin A, GlyA) showed that upon induction of differentiation, CD71 expression decreased, whereas the percentage of GlyA-positive cells increased (Figure 3B), in full agreement with a previous report by Papasavva et al, (2021). The expression of α- and γ-globins, the globin chains expressed by HUDEP-1 cells, was also increased in DM-cultured cells *versus* EM-cultured cells (Figures 3C,D). Accordingly, upon centrifugation, DM-cultured cells gave a red cell pellet (Figure 3E), an indicator of the production of abundant levels of hemoglobin (Kurita et al., 2013).

### 3.4 RPS26 downregulation impairs erythroid differentiation

**FIGURE 4:**
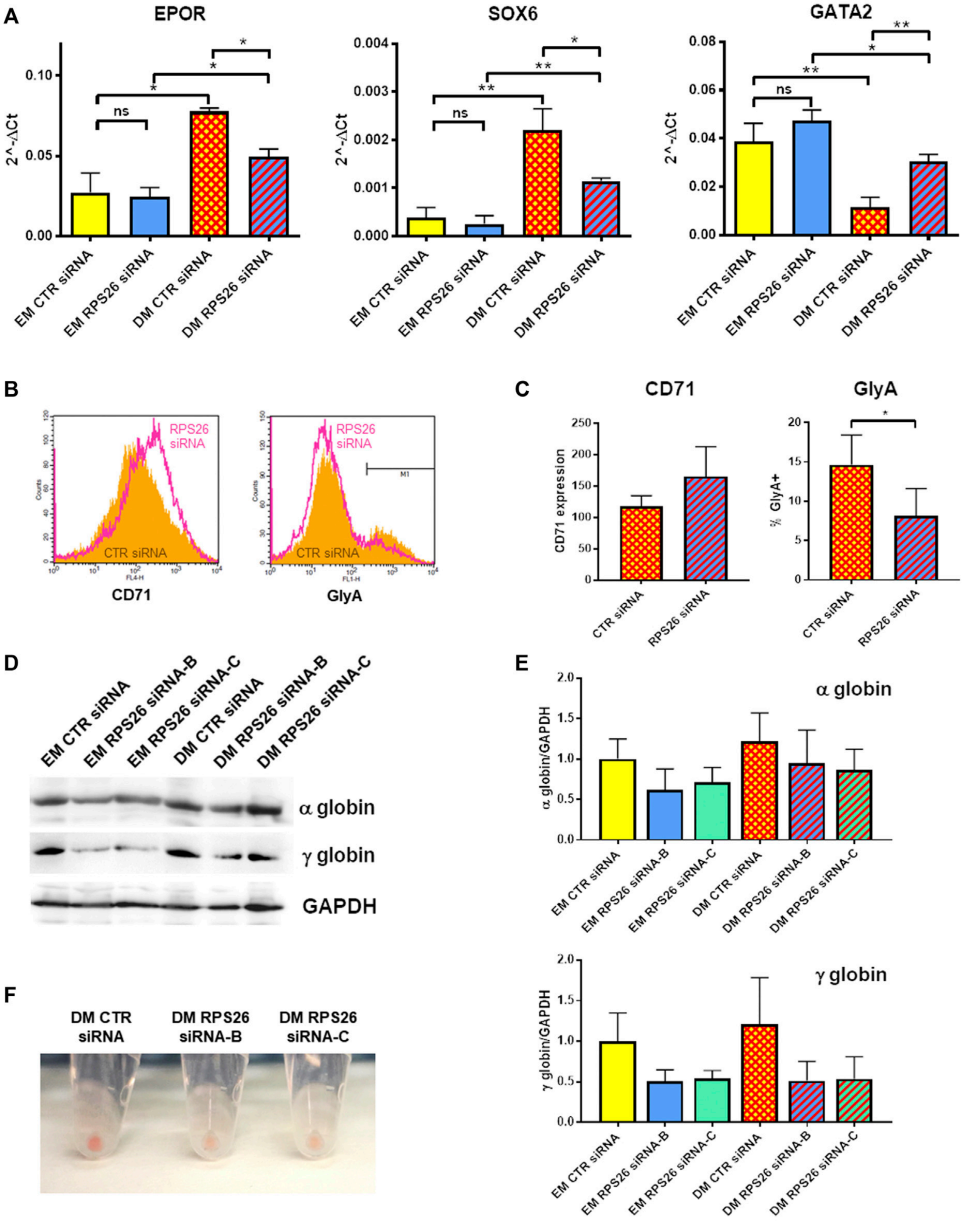
Erythroid differentiation after RPS26 downregulation. **(A)** Expression of erythroid transcripts in DM- and EM-cultured cells treated with control or RPS26-specific siRNAs, evaluated by qRT-PCR. Results from two experiments performed with RPS26 siRNA-B and two experiments performed with RPS26 siRNA-C were normalized to *GAPDH* expression and presented as mean ± SEM; *p ≤ 0.05, **p ≤ 0.01, ns not significant. **(B)** Representative flow cytometry analysis of the erythroid markers CD71 and GlyA in control (orange) and *RPS26*-silenced (pink) cells. For this experiment, RPS26 siRNA-B was used. **(C)** Left: bar graph showing the mean ± SD of the median values of fluorescence for CD71 staining of control and RPS26-silenced cells (two experiments with RPS26 siRNA-B and one experiment with RPS26 siRNA-C). Right: bar graph showing the mean ± SD of the percentage of GlyA-positive cells in control and RPS26-silenced cells (two experiments with RPS26 siRNA-B and one experiment with RPS26 siRNA-C); *p ≤ 0.05. **(D)** Representative immunoblot performed on control and RPS26-silenced cells cultured in EM and DM, showing the expression levels of α- and γ-globins; GAPDH was used as loading control. **(E)** Densitometry analysis performed on three replicates; error bars represent SD. **(F)** Representative images of cell pellets after culturing in DM.

It has been previously described that erythroid differentiation and hemoglobin accumulation are reduced in *RPS19*-deficient cells cultured in presence of EPO (Hamaguchi et al., 2002; Flygare et al., 2005). We therefore investigated how RPS26 downregulation affects erythroid maturation in HUDEP-1 cells cultured in DM. After nucleofection with control or *RPS26*-specific siRNAs, cells were cultured in EM for 2 days and then in either EM or DM for two more days. RPS26-deficient cells cultured in DM (DM RPS26 siRNA) showed decreased levels of *EPOR* and *SOX6* transcripts and increased level of *GATA2* transcript, compared to control cells (DM CTR siRNA. Figure 4A). The expression of these erythroid transcripts was significantly increased in DM-cultured cells, suggesting that RPS26-downregulated cells are indeed capable of erythroid maturation, but with a lower efficiency than control cells (Figure 4A). The changes in *GATA1* expression showed a congruent trend but did not reach statistical significance (Supplementary Figure S2). Anti-CD71 staining and analysis by flow cytometry showed an increased expression of this transmembrane protein in DM RPS26 siRNA cells compared to DM CTR siRNA cells, whereas the percentage of GlyA-positive cells was decreased in DM RPS26 siRNA cells (Figures 4B,C). Western blot analysis showed that the levels of α- and γ-globins were decreased in RPS26-downregulated cells (Figures 4D,E). Finally, the reduced production of hemoglobin in RPS26-deficient cells was clearly visible upon cell centrifugation (Figure 4F). All these results indicate an impaired erythroid differentiation in RPS26-silenced cells.

## 4 Discussion

Patients with DBA display a selective deficiency of erythroid precursors in the bone marrow. The conundrum of tissue specificity in DBA has been addressed using several erythroid cell models but has not been fully solved yet. In experiments evaluating lineage-specific differentiation of *RPS19*-silenced CD34^+^ cells, Dutt and coll. Observed that p53 levels were significantly higher in erythroid cells compared with myeloid cells (Dutt et al., 2011). Moreover, treatment of control CD34^+^ cells with nutlin-3, a p53 activator, selectively impaired erythropoiesis, even in the absence of ribosome dysfunction (Dutt et al., 2011). Another important contributor to the pro-apoptotic phenotype of erythroid DBA cells is the imbalance between globin and heme synthesis due to the reduced number of functional ribosomes (Yang et al., 2016). The consequent accumulation of free heme increases reactive oxygen species production, whereas the inibition of heme synthesis by succinylacetone improves the erythroid differentiation of DBA cells (Yang et al., 2016). Low ribosome concentration not only reduces protein synthesis in general, but also selectively affects the translation of specific transcripts encoding proteins involved in hematopoiesis, including GATA1: this is acknowledged as another mechanism of tissue-specificity (Horos et al., 2012; Ludwig et al., 2014).

Notwithstanding the insight gained for *RPS19*-haploinsufficiency, much less is known about the particular phenotypes of cells depleted for other RP genes involved in DBA etiology. Some reports suggest that mutations in distinct RP genes lead to differences in erythroid phenotypes (Moniz et al., 2012; Gastou et al., 2017) and predisposition to cancer (Lipton et al., 2022). In particular, it is interesting to note that among the most prevalent DBA genes, *RPS26* is the only one that has never been found mutated in patients that developed cancer or myelodysplastic syndrome (Lipton et al., 2022). To explain this peculiarity, it has been hypothesized that RPS26-deficient 40S subunits found in *RPS26*-mutated DBA patients could selectively translate subsets of mRNAs with a protective function against cancer development (Lipton et al., 2022). Recent work has shown that yeast Rps26 exhibits some unique properties among ribosomal proteins, since stresses like high Na^+^ or H^+^ concentrations cause the release of Rps26 from the ribosome *via* binding with the chaperone Tsr2 (Ferretti et al., 2017; Yang and Karbstein, 2022). Rps26-deficient ribosomes preferentially translate specific mRNAs involved in stress-response pathways until the stress subsides and Rps26 is reincorporated into ribosomes (Ferretti et al., 2017). The existence of specialized ribosomes lacking RPS26 in human cells has not been proven yet. We therefore chose to prepare a new DBA model where the effects of RPS26 deficiency could be studied. The early onset of anemia in DBA patients suggests that both fetal and adult erythropoiesis are affected. We silenced *RPS26* in HUDEP-1 cells, an immortalized human cell line that expresses HbF and can be induced to differentiate along the erythroid lineage, to generate a model that recapitulates fetal erythropoiesis in DBA. To our knowledge, this is the first erythroid model of RPS26-deficiency.

Our results show that the depletion of RPS26 by siRNA prevents the proper processing of rRNA, resulting in the decrease of 18S mature rRNA (Figures 1D,E), in agreement with a previous study performed on lymphoblastoid cells with mutated *RPS26* and HeLa cells treated with *RPS26*-siRNAs (Doherty et al., 2010). RPS26-deficient cells showed reduced number of viable cells and increased number of dead cells (Figure 2A). The distribution of cells in different phases of cell cycle was altered, indicating that RPS26 downregulation inhibited proliferation through a G0/G1 phase arrest (Figure 2B). These findings are consistent with other reports obtained on RP-mutated or downregulated models (Farrar et al., 2008; Miyake et al., 2008; Aspesi et al., 2017). In HUDEP-1, knockdown of RPS26 resulted in increased apoptosis, detected by annexin staining (Figures 2C,D), and the treatment with the p53 inhibitor pifithrin−α did not significantly decrease annexin V positivity, suggesting the involvement of p53-independent apoptotic pathways. The level of four genes (*CDKN1A*, *PUMA*, *NOXA*, *TIGAR*) whose expression is induced by p53 stabilization but also by p53-independent mechanisms (Ploner et al., 2008; Yu and Zhang, 2008; Abbas and Dutta, 2009; Tang et al., 2021), was upregulated in RPS26-deficient HUDEP-1 cells. A previous study performed on a human fibroblast cell line showed that *RPS26*-specific siRNAs increased the levels of p53 protein and its target genes, and that simultaneous knockdown of RPS26 and p53 restored, at least partially, both the expression of p53 target genes and cell growth (Cui et al., 2014). Conversely, the same study reported that RPS26 knockdown in a colon cancer cell line had minimal or no effect on p53 levels and its target genes (Cui et al., 2014). Furthermore, deficiency of other RPs leads to the activation of p53-independent responses (Torihara et al., 2011; Aspesi et al., 2014; Singh et al., 2014), and it is still unclear which pathways are predominant in human erythroid progenitors.

Of interest, our data show that RPS26-deficient cells have an increase of the basal level of cytoplasmic calcium (Figure 2F). To our knowledge, this is the first indication of impaired calcium homeostasis in DBA, whereas high intracellular calcium concentration levels have been already described in lymphoblast cell lines derived from patients with Shwachman-Diamond syndrome (Ravera et al., 2016), another ribosomopathy characterized by increased stabilization of p53 (Warren, 2018). The observation that inhibitors of the calcium-binding protein calmodulin improve p53-mediated apoptosis and defective erythropoiesis both in zebrafish *rps29* mutant embryos and in *RPS19*-silenced CD34^+^ cells, support the involvement of calcium in determining the altered phenotype of DBA cells (Taylor et al., 2020). However, treatment with the Ca^2+^ chelator EGTA did not reduce annexin V positivity in our RPS26-deficient model (not shown), possibly because either the inhibition of calcium accumulation was insufficient to attenuate apoptosis, or other calcium-independent pathways were involved in cell death induction. Altogether, these data suggest that *RPS26* silencing leads to cells growth inhibition and apoptosis, likely through p53-independent signaling mechanisms.

Primary erythroid cells from DBA patients are scarce and seldom available for research purpose, therefore studies are routinely performed on non-erythroid DBA cells (Choesmel et al., 2007; Robledo et al., 2008; Aspesi et al., 2017). The advantages of using HUDEP-1 cells to model DBA (Wang et al., 2022) is that they can be easily induced to differentiate into more mature erythroid stages and that their handling is less laborious and expensive than establishment of patient-derived induced Pluripotent Stem Cells (iPSC) or RP downregulation in hematopoietic cells isolated from healthy donors. HUDEP-1 cells cultured in DM for 2 days displayed increased expression of erythroid transcripts (Figure 3A), such as *EPOR*, *GATA1* and the marker of definitive erythropoiesis *SOX6* (Yi et al., 2006). Flow cytometry analysis showed a reduction of CD71 and an increase of GlyA membrane proteins after culture in DM, whereas immunoblotting showed increased globin levels (Figures 3B,C). When subjected to *RPS26*-silencing, cells showed impaired erythroid differentiation, as assessed by expression analysis of erythroid markers and globin levels (Figures 4A–E). This suggests that *RPS26*-deficiency impairs or delays erythroid maturation, similarly to what already reported for *RPS19*- mutated or downregulated models (Hamaguchi et al., 2003; Flygare et al., 2005; Moniz et al., 2012; Garçon et al., 2013).

In conclusion, *RPS26*-deficient HUDEP-1 cells show defective rRNA maturation, increased apoptosis, and impaired erythropoiesis. For the first time, we have obtained and characterized a new erythroid model that recapitulates the phenotype of DBA cells with pathogenic variants in *RPS26* and that can be used to study DBA pathophysiology.

## Data Availability

The original contributions presented in the study are included in the article/Supplementary Material, further inquiries can be directed to the corresponding author.
