# Supplementary material for: Deficiency of ribosomal protein S26, which is mutated in a subset of patients with Diamond Blackfan anemia, impairs erythroid differentiation
Source: Front Genet. 2022 Dec 12;13:1045236. doi: 10.3389/fgene.2022.1045236 (PMC9790993; doi:10.3389/fgene.2022.1045236)
Supplement: Supplementary file 1 [file DataSheet1.docx]

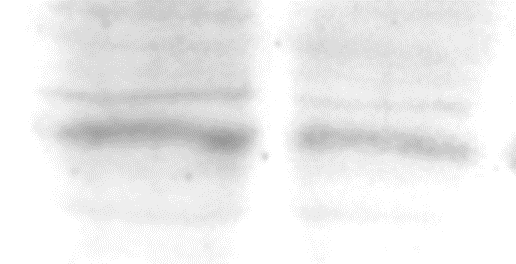

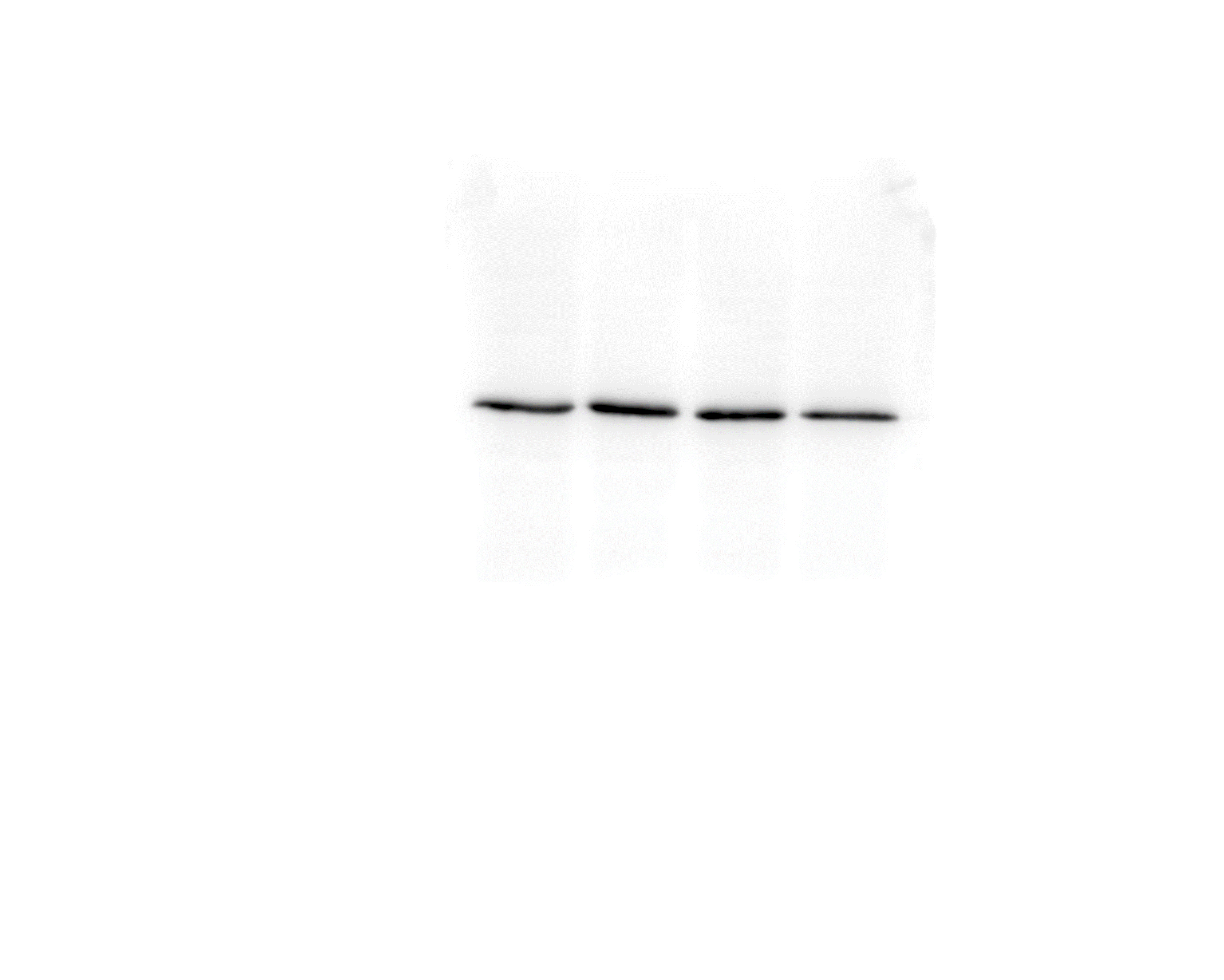


**CTR siRNA**

**RPS26 siRNA-B**

**GAPDH**

**p53**

**Fig. S1.** **Expression of p53 protein in RPS26-silenced HUDEP1 cells.**

Representative immunoblot performed on HUDEP1 cells treated with siRNAs and cultured for 4 days in expansion medium.

**Fig. S2.** **Expression levels of *GATA1* transcript in DM-cultured cells.**

*GATA1* expression was evaluated by qRT-PCR in cells treated with control or RPS26-specific siRNAs and cultured in EM or DM. Results from two experiments performed with RPS26 siRNA-B and two experiments performed with RPS26 siRNA-C are shown. Data are normalized on *GAPDH* levels. * p≤0.05, ns not significant

**Supplementary Material**
